# The reliability of the adverse childhood experiences questionnaire for late postpartum use in relation to maternal emotional health

**DOI:** 10.3389/fpsyt.2026.1914194

**Published:** 2026-10-19

**Authors:** Aimé Ozuna, Panteha Hayati Rezvan, Jenny Legido, Sriamsha Dubbaka, Pat Levitt, Kameelah Gateau

**Affiliations:** 1 Developmental Neuroscience and Neurogenetics Program, Children's Hospital Los Angeles, Los Angeles, CA, United States; 2 The Saban Research Institute, Children's Hospital Los Angeles, Los Angeles, CA, United States; 3 Division of Neurology, Children's Hospital Los Angeles, Los Angeles, CA, United States; 4 Keck School of Medicine of the University of Southern California, Los Angeles, CA, United States; 5 Department of Pediatrics, Children’s Hospital Los Angeles, Los Angeles, CA, United States; 6 Department of Pediatrics, Division of Neonatology, Los Angeles, CA, United States

**Keywords:** ACEs (adverse childhood experiences), maternal emotion, patient health questionnaire-9, perceived stress scale, postpartum depression (PPD), test-retest

## Abstract

**Background:**

Childhood Experiences (ACEs) have been linked to poor mental and physical health outcomes in adulthood. The Adverse Childhood Experiences Questionnaire (ACEQ) is commonly used to screen ACEs in many populations. Postpartum women represent a psychologically vulnerable population, possibly impacting recall of ACEs and utility of ACEQ in prognosticating future health problems. In this secondary analysis, we assessed test-retest reliability of the ACEQ among postpartum women, also screened for depressive symptoms and perceived stress.

**Methods:**

317 mother-infant dyads from a larger longitudinal study completed the deidentified ACEQ, Patient Health Questionnaire-9 (PHQ-9), and Perceived Stress Scale (PSS) at approximately 6 and 12 months postpartum. Test-retest reliability was assessed using Intraclass Correlation Coefficient (ICC) with two-way mixed-effects models; absolute agreement ICC evaluated score stability, a consistency ICC sensitivity analysis assessed systematic bias.

**Results:**

The ACEQ demonstrated good test-retest reliability (absolute agreement ICC = 0.79, *p* < 0.001), with a nearly identical ICC consistency suggesting no systematic bias. Higher baseline PSS and PHQ-9 were associated with higher ACEQ scores at both timepoints. Although nearly half of mothers reported different ACEQ scores at 12 months, most changes fell within measurement error, and reporting discrepancy was not associated with baseline emotional distress.

**Discussion:**

The de-identified ACEQ is a reliable measure of early adversity during the first year postpartum, though its limited sensitivity to small individual change should be considered when tracking scores over time. Its reliability earlier in the postpartum period or perinatally, when depression is more prevalent and screening is most valuable, warrants future study before clinical application.

## Introduction

Experiencing early life adversity (ELA), including mental, emotional and sexual abuse, and household dysfunction, has been linked to significant mental and physical health morbidities in adulthood (1). The Adverse Childhood Experiences Questionnaire (ACEQ) is an assessment tool utilized regularly by healthcare institutions, such as Kaiser Permanente, to screen for ELA (2). The ACEQ is a retrospective questionnaire in which respondents recall traumatic events that occurred during their first 18 years after birth. At the population level, the ACEQ has been associated with a range of adverse health outcomes, including cardiovascular disease (ischemic heart disease, stroke), metabolic conditions (diabetes), and cancer (3–5). Accurate characterization of ELA experiences is of particular importance during the perinatal period, during which birthing parents with high ELA exposure have an increased risk of experiencing postpartum depression (PPD) (6). Perinatal depression, which occurs during pregnancy and up to one-year postpartum, affects approximately 1 in 7 individuals and is characterized by persistent sadness, low self-esteem, sleep disruption, anxiety, and difficulties bonding with the baby (7). However, there are limitations in ACEQ’s ability to predict health morbidities at the individual level (8, 9). Previous studies suggest that, given the retrospective nature of the ACEQ, recall bias and respondents’ emotional state at the time of administration may impact the ability to accurately report ELA (8, 9), thus limiting its ability to predict future health outcomes. The ability to stratify mothers at the highest risk of postpartum mood and he`alth disorders requires reliable screening measures. To the authors’ knowledge, no published studies have examined the reliability of the ACEQ among pregnant or postpartum women, including those experiencing postpartum mood disorders or physiological health diagnoses. The present study begins to address this gap by examining the reliability of the ACEQ and its relationship with maternal emotional status during the first postpartum year.

Reliability, commonly used synonymously with agreement, is defined as an evaluation of variability between two instances (10); in this study, reliability evaluates variability in scores among the same subjects between two timepoints. The reliability of the ACEQ was established in the original study population, comprising 9,508 adults, ages 19-65+, with 80% identifying as White, 6% identifying as Asian, 5% identifying as Black, 5% identifying as Hispanic/Latine, and 4% identifying as an unlisted race (1). Among the original population, approximately 52% of the sample identified as women. Follow up studies have since evaluated reliability among other populations, including children, adolescents, college students, and individuals experiencing mental health conditions. Across these populations, reliability ranged from moderate to excellent, with Pearson’s r ranging from 0.71-0.91 and Intraclass Correlation Coefficient (ICC) ranging from 0.88-0.91 (11–16). However, some studies suggest that reliability may be influenced by the participant’s emotional state at the time of administration and by the interval between administrations (12, 17). Colman et al. (17) found that participants experiencing depression or psychological distress were more likely to report previously unreported adverse childhood experiences, raising questions about whether emotional state systematically influenced ACEQ responses.

Recent evidence suggests that ELA may have a strong impact on postpartum health and emotional well-being. For example, Aydogan et al., 2025 reported that ELA may act as a primary risk factor for postpartum emotional state, directly increasing vulnerability to mental illness (18). Their study evaluated associations between ACEs and PPD development, accounting for birth trauma, and found that birthing parents with higher ACE scores faced an increased risk of PPD. Similarly, Prentice et al. identified a correlation between greater ACEs exposure and increased incidences of PPD;emphasizing the importance of screening during the peripartum period, given that “mental health conditions including suicide, overdose, and unintentional injuries [being] a leading cause of death in all pregnant patients” (19).

Perinatal mood disorders are prevalent and impact the health and well-being of both mother and infant. Given the impact of PPD on maternal and infant developmental outcomes and the potential for ELA screening to identify modifiable risk factors, determining the reliability of the ACEQ in postpartum populations is essential. Reliable ACEQ screening can guide clinical care and early intervention to mitigate or prevent downstream health perturbations. In this study, we examine the test-retest reliability of the ACEQ among postpartum women, the majority self-identified ethnicity as Hispanic, at approximately 6 and 12 months post-delivery.

To this end, the present study aimed to: (1) evaluate the test-retest reliability of the ACEQ between 6 and 12 months postpartum; (2) determine whether early postpartum emotional status – perceived stress and depressive symptoms – was associated with (a) reported ACEQ scores over time and (b) the likelihood of reporting discrepancy between visits; and (3) characterize the direction of change in reported ACEQ scores and factors associated with it.

## Methods

### Study participants

A total of 340 mothers were enrolled from a pool of 789 eligible postpartum women as part of a parent longitudinal study, The Family First Research Study, which aims to assess the impact of ELA on infant and toddler neurodevelopment. Participants were recruited as mother-infant dyads, primarily through a partnership with Kaiser Permanente in the greater Los Angeles area of Southern California. Mothers were eligible to enroll in the study if they were over the age of 18, were the biological parent of the participating child, and spoke primarily English or Spanish. Participants were not screened for depression or stress symptoms at the time of enrollment but were screened for history of developmental or psychiatric disorders among any of the child’s first-degree relatives, which would result in the dyad’s exclusion. Enrollment occurred within 6 months of the child’s birth. Non-enrollment of the remainder of the eligible pool resulted from non-responsiveness after screening was completed, scheduling conflicts, or a change in interest to participate.

Data for this study were collected at two timepoints: a baseline visit at approximately 6 months postpartum, and a follow-up visit at approximately 12 months postpartum. At each visit, mothers completed the 10-item ACEQ to screen for early adversity experienced during the first 18 years of life. Mothers also completed the Perceived Stress Scale (PSS) and the Patient Health Questionnaire (PHQ-9) to assess stress levels and depression symptoms at each visit. A final total of 317 mothers completed the ACEQ at both timepoints, qualifying them for inclusion in the analytic sample for this study. **Figure 1** illustrates a visual representation of the participant selection process.

**Figure 1:**
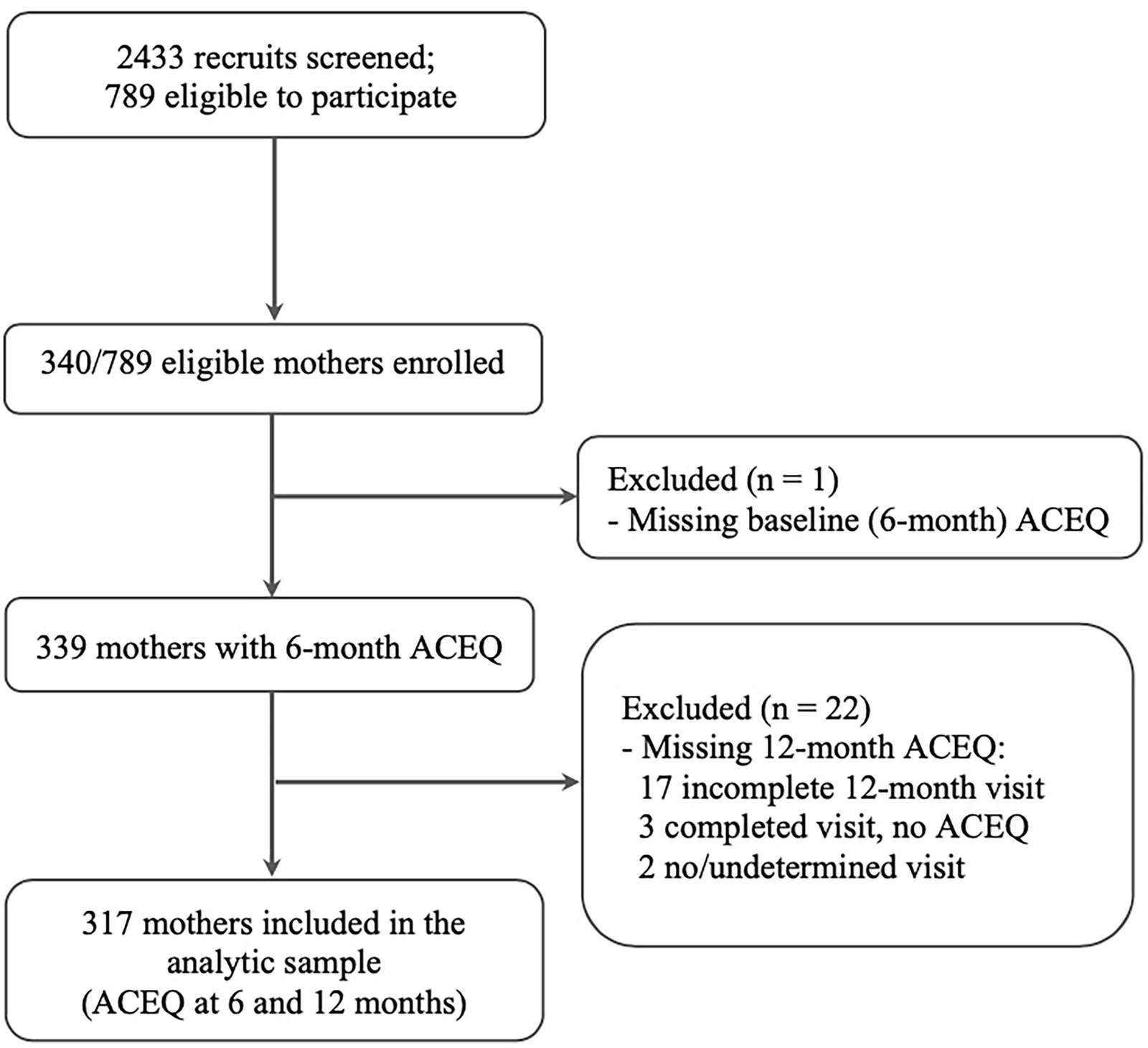
Participants flow diagram. Of 2,433 recruits screened for eligibility in the Family First Research Study, 789 were deemed eligible and 340 enrolled. Of the 340 enrolled mothers, all of whom completed a 6-month visit, 1 was excluded for a missing 6-month ACEQ score, yielding 339 with baseline ACEQ data. An additional 22 were excluded for missing 12-month ACEQ scores: 17 with an incomplete 12-month visit, 3 who completed the visit but did not have ACEQ recorded, and 2 with no or undetermined visit status, resulting in a final analytic sample of 317 mothers with ACEQ scores at both timepoints. ACEQ, Adverse Childhood Experiences Questionnaire.

### Measurements

#### Adverse childhood experiences questionnaire

The ACEQ was administered at 6 and 12 months postpartum, assessing exposure to 10 categories of childhood trauma including abuse, neglect, and household dysfunction. The ACEQ is a tool originally developed by a group of medical investigators at the Kaiser Permanente San Diego Health Appraisal Clinic, as part of the Southern California Permanente Medical Group, in an effort to characterize exposure to childhood adversity as a risk factor for increased risk of negative health outcomes in adulthood (1). The questionnaire format utilized for this study was de-identified to minimize the risk of mothers’ reluctance to endorse sensitive questions accurately and reliably. Participants were asked to read through the 10 questionnaire items and report a total number of items they felt were an accurate representation of their experiences of adversity in childhood and adolescence. As a result, internal consistency (e.g., Cronbach’s α) for this questionnaire could not be evaluated, as individual item endorsements were not recorded.

Total ACEQ scores range from 0 to 10, with one point for each type of trauma experienced, and higher scores to indicate greater cumulative adversity. Risk levels were categorized as “Low” (0), “Intermediate” (1 – 3), or “High” (4 – 10). Discrepancy in reported ACEQ scores was characterized using a binary variable indicating whether participants reported different ACEQ scores at 6 and 12 months postpartum (1 = any change, 0 = identical scores). A three-level categorical variable classifying participants as: (1) stable (ACEQ_6 months_ = ACEQ_12 months_), (2) increased (ACEQ_12 months >_ ACEQ_6 months_), or (3) decreased (ACEQ_12 months_< ACEQ_6 months_) was used to characterize the direction of change in reporting ACEQ scores. Participants reporting zero ACEs at baseline could not report decreases at follow-up, creating a floor effect that constrains the direction of possible score change among low-risk participants.

#### Perceived stress scale

Perceived stress was assessed using the PSS, a 10-item instrument rated on a Likert scale from 0 (“Never”) to 4 (“Very often”) (20). Scores range from 0 to 40, with higher scores indicating greater perceived stress. We dichotomized scores as “Low stress” (0 – 13) versus “Moderate – High stress” (14 – 40). This questionnaire was utilized to determine severity of stress symptoms experienced by the reporting individual. Participants were asked at both timepoints to report the frequency, if any, of the symptoms in the questionnaire within the last 30 days. This questionnaire was administered at all research visits within the larger research study from which this data was drawn. In the current study sample, PSS demonstrated good internal consistency (Cronbach’s α = 0.85).

#### Patient health quetionnaire-9

Depressive symptoms were evaluated using the PHQ-9, a module developed and validated from the Primary Care Education of Mental Disorders (PRIME-MD) (21).

The PHQ-9 is a 9-item screening tool rated on a Likert scale from 0 (“Not at all”) to 3 (“Nearly every day”), with higher scores indicating greater depressive symptoms. We classified scores as “No depression” (0 – 4) versus “Mild – Severe depression” (5 – 27). While PHQ-9 is typically categorically rated using a standard five-tier PHQ-9 classification (none/minimal, mild, moderate, moderately severe, severe), the distribution of PHQ-9 scores in this study sample was heavily skewed towards minimal or zero-value scores. Given the small number of participants in the higher severity ranges, finer categorization would have produced insufficient cell sizes for reliable statistical comparison. We therefore dichotomized the sample using the established clinical threshold of ≥5, which distinguishes participants with any clinically relevant depressive symptoms from those without. Importantly, the PHQ-9 was also examined as a continuous predictor in all models, preserving the full range of scores and addressing the concern about loss of clinical information. Participants were asked at both timepoints to report the frequency at which they experienced listed symptoms within the previous two weeks. The internal consistency of PHQ-9 in the current study sample was good (Cronbach’s α = 0.84).

While the Edinburgh Postnatal Depression Scale (EPDS) is widely regarded as a gold standard in specialty care for assessing postpartum depression risk, the PHQ-9 assessment was implemented in the parent longitudinal study protocol with the goal of quantifying depressive symptoms and characterizing depression symptom severity at the time of participation. Past publications have highlighted that the PHQ-9 language directly assesses the 9 diagnostic criteria for major depressive disorder (MDD) (22) as established in the Diagnostic and Statistical Manual of Mental Disorders (DSM), thus the PHQ-9 was implemented given its generalizability and adherence to DSM criteria (23). Additionally, the assessment is listed by the American Psychiatric Association as a recommended clinical resource for initial assessment of depression-specific severity (23), supporting its value as an ideal assessment tool. Finally, the Family First Study included a large number of questionnaires, and thus the PHQ-9 was selected as the sole depression assessment with consideration for the burden for having two instruments to identify depressive symptoms at 6 and 12 months rather than one.

### Statistical analysis

All analyses were restricted to mothers with reported ACEQ scores at both 6 and 12 months postpartum.

Test-retest reliability of the ACEQ was assessed using Intraclass Correlation Coefficient (ICC) with two-way mixed-effects models (10). The primary analysis used absolute agreement ICC to evaluate stability of reported ACEQ scores between 6 and 12 months postpartum (24). A sensitivity analysis using consistency ICC was performed; comparison of the absolute-agreement and consistency estimates indicates the extent to which systematic bias between timepoints affects reliability. Cohen’s kappa was calculated to assess categorical agreement, with reported ACEQ scores classified into ACE risk categories (“Low”: 0 ACEs, “Intermediate”: 1–3 ACEs, “High”: 4+ ACEs) (25, 26). Standard error of measurement (SEM) and minimum detectable change at 95% confidence (MDC_95_) were calculated to quantify measurement precision and the threshold for detecting true individual change (27–29). SEM was calculated as SD×√(1−ICC), where SD is the pooled standard deviation across both timepoints; SEM represents the standard deviation of measurement error. MDC_95_ was calculated as 1.96×√2×SEM, representing the smallest change that exceeds measurement error with 95% confidence. Agreement between timepoints was further assessed using Bland-Altman analysis, calculating the mean difference (bias) and 95% limits of agreement. Proportional bias was evaluated by regressing the differences between timepoints on their mean, where a non-significant slope indicates that agreement does not depend on ACEQ score magnitude (30, 31).

Spearman rank correlations were used to examine bivariate associations between baseline emotional distress, including perceived stress (PSS), depressive symptoms (PHQ-9), and reported ACEQ scores at 6 and 12 months postpartum. Linear mixed-effects models with restricted maximum likelihood estimation were conducted to: (1) examine whether reported ACEQ scores changed from 6 to 12 months postpartum, and (2) assess whether baseline emotional distress (PSS and PHQ-9) at 6 months was associated with reported ACEQ scores across timepoints. We fit a series of models, all including random intercepts for participants to account for within-person correlation. Separate models assessed associations between baseline symptoms and reported ACEQ scores. Both PSS and PHQ-9 were examined as continuous scores and as categorical classifications using the categories defined above, with each model including fixed effects of time and the baseline symptom measure. We tested interactions between time and each symptom measure (both continuous and categorical) to determine whether associations differed across timepoints. Residual diagnostics, including quantile-quantile plots of residuals and random effects and residual-versus- fitted plots, were performed to assess distributional assumptions and model fit. Models were refit with robust standard errors to evaluate sensitivity of results to potential heteroscedasticity.

Baseline reported ACEQ scores and emotional distress measures were compared between participants whose reported ACEQ scores changed (discrepancy group) and those whose scores did not change (no change group). Comparisons were made with chi-square tests for categorical variables and Wilcoxon rank-sum tests for continuous variables, given their skewed distributions. To examine factors associated with any change in reported ACEQ scores between 6 and 12 months postpartum, we conducted logistic regression models with discrepancy (any change vs. no change) as the outcome. Baseline ACEQ score, ACE risk level, and emotional distress measures, including perceived stress (PSS) and depressive symptoms (PHQ-9), were examined in separate unadjusted models. Because baseline ACEQ score is itself part of the change-based outcome, we adjusted for it in sensitivity analyses, entering it as a covariate in continuous (linear and quadratic) and categorical forms. Both PSS and PHQ-9 were examined as continuous scores and as categorical classifications in separate models.

Baseline reported ACEQ scores and emotional distress measures were further compared across mothers with stable, increased, and decreased reported ACEQ scores using chi-square tests for categorical variables and Kruskal-Wallis tests for continuous variables. To examine factors associated with the direction of change, we conducted multinomial logistic regression with change direction (stable [reference category], increased, decreased) as the outcome, following the same modeling approach used for discrepancy. We also conducted an exploratory analysis restricted to mothers with at least one baseline ACE (n = 208), among whom decreases were possible.

For logistic and multinomial regression models, goodness-of-fit was assessed using the Hosmer-Lemeshow test, and multicollinearity was assessed using variance inflation factors. Linearity of the logit was assessed for continuous covariates; where it was not supported, baseline ACEQ was modeled using the quadratic and categorical specifications described above. Statistical significance was defined *a priori* as a two-sided p-value< 0.05. Analyses were performed using Stata/MP version 19 (StataCorp LLC, College Station, TX).

## Results

### Characteristics of the study participants

Of 340 eligible mothers at 6 months postpartum, 317 (93.2%) completed the ACEQ at both 6 and 12 months and were included in the analysis (**Figure 1**). Demographic characteristics at baseline are summarized in **Table 1**. The sample was predominantly Hispanic (66.2%, n = 200) and White (67.3%, n = 150). The mean maternal age was 32.8 years (SD = 5.3). Most participants were married (62.5%, n = 192), and over half had a bachelor’s degree or higher (56.8%, n = 171). Two-thirds reported annual family incomes of ≥ $50,000 (67.0%, n = 175). Regarding employment, 42.3% (n = 129) were employed full-time and an additional 29.5% (n = 90) were homemakers/raising children full-time. At baseline, the median PSS score was 12 (IQR: 7 – 16), and 39.7% (n = 126) reported moderate-to-high stress. The median PHQ-9 score was 2 (IQR: 0 – 5), and over one-quarter (26.8%, n = 85) reported mild-to-severe depressive symptoms. The median ACEQ score was 1 (IQR: 0 – 3). Based on risk levels, 41.3% (n = 131) had intermediate-risk ACEs (1 – 3) and 24.3% (n = 77) had high-risk ACEs (4+), which is substantially greater than the reported prevalence in the most recent CDC national data report (32).

**Table 1:** Demographics and clinical characteristics of mothers at 6 months postpartum (n = 317).

Demographics	Mean (SD) and median [25th, 75th percentiles]
Age (years)	32.8 (5.3)
	33.0 [28.9, 37.0]
Ethnicity	
Non-Hispanic	102 (33.8%)
Hispanic	200 (66.2%)
Race	
White	150 (67.3%)
Black or African American	21 (9.4%)
American Indian or Alaska Native	7 (3.1%)
Asian	29 (13.0%)
Native Hawaiian or Pacific Islander	2 (0.9%)
Multiple races	14 (6.3%)
Marital status	
Single	62 (20.2%)
Married	192 (62.5%)
Cohabitating	46 (15.0%)
Widowed/Separated/Divorced	7 (2.3%)
Highest level of education	
< High school (HS) Diploma	14 (4.7%)
HS Diploma/GED	71 (23.6%)
Associates Degree	45 (15.0%)
Bachelor's Degree	86 (28.6%)
Postgraduate Degree	85 (28.2%)
Employment status	
Full-time employed	129 (42.3%)
Part-time employed	38 (12.5%)
Unemployed/Laid off	14 (4.6%)
Homemaker/Raising children full-time	90 (29.5%)
On maternity leave	34 (11.1%)
Annual household income	
> $50,000	86 (33.0%)
$50,000 -$99,999	77 (29.5%)
$100,000 or greater	98 (37.5%)
Maternal emotional state, and early life adversity	
PSS total score	11.9 (6.3)
	12.0 [7.0, 16.0]
PSS level	
Low	191 (60.3%)
Moderate-High	126 (39.7%)
PHQ-9 total score	3.2 (3.6)
	2.0 [0.0, 5.0]
PHQ-9 severity of depressive symptoms	
None	232 (73.2%)
Mild-Severe	85 (26.8%)
ACEQ total score	2.2 (2.5)
	1.0 [0.0, 3.0]
ACEs risk level	
Low	109 (34.4%)
Intermediate	131 (41.3%)
High	77 (24.3%)
ACEQ reporting discrepancy	
No change	167 (52.7%)
Any change	150 (47.3%)
Direction of discrepancy in ACEQ reporting	
Decreased	72 (22.7%)
Stable	167 (52.7%)
Increased	78 (26.6%)

Data are frequency and percentage, mean (SD) and median [25^th^, 75^th^ percentiles]. The frequency and percentage of incomplete/not disclosed data: Ethnicity (n = 15, 4.7%); Race (n = 94, 29.7%); Marital status (n = 10, 3.2%); Education (n = 16, 5.1%); Employment (n = 12, 3.8%); Annual family income (n = 56, 17.7%).

### Reliability

The ACEQ demonstrated good test-retest reliability (**Table 2**). The absolute agreement ICC was 0.79 (95% CI: 0.75, 0.83, *p* < 0.001), indicating 79% of the variance in ACEQ scores reflected true between-person differences rather than measurement error. The consistency ICC was nearly identical (ICC = 0.79, 95% CI: 0.75, 0.83, *p* < 0.001), suggesting no systematic bias between timepoints. Cohen’s kappa (κ) for categorical agreement was 0.62 (95% CI: 0.54, 0.70; *p* < 0.001), indicating substantial agreement across ACEs risk categories. A linear mixed-effects model revealed no significant evidence that reported ACEQ scores systematically changed from 6 to 12 months postpartum (*β* = 0.04, 95% CI: -0.13, 0.22; *p* = 0.626), further confirming an absence of systematic bias and indicating overall population-level stability. This conclusion was unchanged when the model was refit with robust standard errors to account for the mild heteroscedasticity observed in residual diagnostics. The SEM was 1.12, yielding a MDC_95_ of 3.10 points (31.0% of the scale range), suggesting limited sensitivity to detect small individual changes. This indicates that individual-level changes of 3.10 points or more exceed measurement error with 95% confidence and likely represent true change rather than measurement variability. Examination of individual-level changes revealed high stability. Of 317 participants, 167 (52.7%) reported identical ACEQ scores at both timepoints, and a further 136 (42.9%) changed by less than the MDC_95_ (< 3.10 points); thus 303 (95.6%) were stable within measurement error. Only 14 (4.4%) showed change exceeding the MDC_95_ threshold – 7 (2.2%) decreases and 7 (2.2%) increases – consistent with true individual change. Bland-Altman analysis showed a mean difference of 0.04 (95% CI: -0.13, 0.22), indicating no systematic bias between timepoints. The 95% limits of agreement ranged from -3.12 to 3.20, closely corresponding in magnitude to the MDC_95_ of 3.10. Regression of the difference on the mean showed no significant proportional bias (*β* = 0.04, 95% CI: -0.03, 0.12; *p* = 0.245), indicating that agreement did not vary with ACEQ score magnitude (**Supplementary Figure S1**).

**Table 2:** Test-retest reliability of the ACEQ between 6 and 12 months postpartum.

Statistic	Estimate	(95% CI)	*p-*value
ICC, absolute agreement	0.79	(0.75, 0.83)	**< 0.001**
ICC, consistency	0.79	(0.75, 0.83)	**< 0.001**
Cohen’s κ (risk categories)	0.62	(0.54, 0.70)	**< 0.001**
Change in reporting over time (*β*)^†^	0.04	(-0.13, 0.22)	0.626
SEM	1.12	–	–
MDC_95_	3.10	–	–

^†^ Fixed effect of time (12 vs. 6 months) from a linear mixed-effects model, reflecting population-level stability. *p* < 0.05 is shown in bold font.

ICC, Intraclass correlation coefficient; MDC95, Minimal detectable change at 95% confidence.

### Associations between ACEQ scores and baseline emotional distress

Baseline emotional distress (6 months postpartum) was positively correlated with ACEQ scores at both timepoints (**Supplementary Table S1**). Associations between baseline PHQ-9 and ACEQ scores were consistent across timepoints (*ρ =* 0.34, *p* < 0.001 at 6 months; *ρ =* 0.31*, p* < 0.001 at 12 months), while the association between baseline PSS and ACEQ scores, though still statistically significant, was attenuated at 12 months (*ρ =* 0.21, *p<* 0.001 at 6 months; *ρ =* 0.12, *p =* 0.03 at 12 months). Baseline PSS and PHQ-9 were moderately correlated with each other (*ρ =* 0.57, *p* < 0.001). Linear mixed-effects models were used to examine whether baseline emotional distress was associated with reported ACEQ scores across timepoints (**Table 3**). When examined continuously, each one-unit increase in baseline PSS was associated with 0.05 additional ACEs reported (95% CI: 0.01, 0.09; *p* = 0.018), and each one-unit increase in baseline PHQ-9 was associated with 0.21 additional ACEs reported (95% CI: 0.14, 0.27; *p* < 0.001). When examined categorically, mothers with mild-to-severe depressive symptoms reported significantly more ACEs than those with no depressive symptoms across both timepoints (*β* = 1.45, 95% CI: 0.88, 2.02; *p* < 0.001), while the difference between the moderate-to-high and low stress groups was not statistically significant (*β* = 0.48, 95% CI: -0.06, 1.01; *p* = 0.08). The interaction terms between baseline emotional distress and timepoint were not significant in any model (all *p* > 0.13), indicating that emotional distress was not associated with the temporal stability of ACEQ reporting between 6 and 12 months postpartum.

**Table 3:** Associations between baseline emotional distress and reported ACE scores: Main and interaction effects from linear mixed-effects models.

	PSS Model				PHQ-9 Model			
	Coef.	SE	(95% CI)	*p*-value	Coef.	SE	(95% CI)	*p*-value
Main effect model, continuous emotional state								
12-month assessment (vs. 6-month)	0.04	0.09	(-0.13, 0.22)	0.626	0.04	0.09	(-0.13, 0.22)	0.626
Baseline emotional state	0.05	0.02	(0.01, 0.09)	**0.018**	0.21	0.03	(0.14, 0.27)	**< 0.001**
Main effect model, categorical emotional state								
12-month assessment (vs. 6-month)	0.04	0.09	(-0.13, 0.22)	0.626	0.04	0.09	(-0.13, 0.22)	0.626
Moderate – high stress (vs. Low)	0.48	0.27	(-0.06, 1.01)	0.080	**-**	**-**	**-**	**-**
Mild – severe depressive symptoms (vs. None)	**-**	**-**	**-**	**-**	1.45	0.29	(0.88, 2.02)	**< 0.001**
Interaction model, continuous emotional state								
12-month assessment (vs. 6-month)	0.300	0.19	(-0.08, 0.68)	0.121	0.047	0.120	(-0.19, 0.28)	0.693
Baseline emotional state	0.061	0.02	(0.02, 0.10)	**0.007**	0.206	0.04	(0.13, 0.28)	**< 0.001**
Baseline emotional state × 12-month assessment	-0.02	0.01	(-0.05, 0.01)	0.134	-0.001	0.03	(-0.05, 0.05)	0.966
Interaction model, continuous emotional state								
12-month assessment (vs. 6-month)	0.14	0.12	(-0.09, 0.36)	0.243	0.05	0.11	(-0.16, 0.26)	0.626
Moderate – high stress (vs. Low)	0.59	0.29	(0.03, 1.15)	**0.039**	**-**	**-**	**-**	**-**
Moderate – high stress (vs. Low) × 12-month assessment (vs. 6-month)	-0.23	0.18	(-0.59, 0.13)	0.211	**-**	**-**	**-**	**-**
Mild – severe depressive symptoms (vs. None)	**-**	**-**	**-**	**-**	1.46	0.31	(0.86, 2.06)	**< 0.001**
Mild – severe depressive symptoms (vs. None) × 12-month assessment (vs. 6-month)	**-**	**-**	**-**	**-**	-0.03	0.20	(-0.43, 0.37)	0.890

All models included random intercepts for participants. Baseline emotional state was measured at 6 months. The main effect of the 12-month assessment reflects population-level stability and is also reported in **Table 2**. PSS, Perceived Stress Scale; PHQ-9, Patient Health Questionnaire-9; Coef., coefficient; SE, standard error; CI, confidence interval. Dashes indicate terms not applicable to that model. *p* < 0.05 is shown in bold font.

### Factors associated with ACEQ reporting discrepancy

Of 317 mothers, 150 (47.3%) reported different ACEQ scores at 12 months compared to 6 months (**Table 1**). Mothers with any reporting discrepancy had higher baseline ACEQ scores than those with stable reporting (median [IQR]: 2.0 [1.0, 4.0] vs. 0.0 [0.0, 2.0]; *p* < 0.001; **Supplementary Table S2**). Reporting discrepancy varied by baseline ACE risk level (χ^2(2)^ = 47.12, *p* < 0.001): 21.1% of low-risk mothers (no ACEs), 58.0% of intermediate-risk mothers (1–3 ACEs), and 66.2% of high-risk mothers (4+ ACEs) showed changes in reported scores. Because the continuous baseline ACEQ-discrepancy association was non-linear (significant quadratic term, *p* < 0.001), this association is presented using ACE risk categories rather than a single per-unit odds ratio: compared to low-risk mothers, those at intermediate risk had 5.17 times higher odds of discrepancy (95% CI: 2.90, 9.19; *p* < 0.001) and those at high risk had 7.33 times higher odds (95% CI: 3.79, 14.18; *p* < 0.001; **Table 4**).

**Table 4:** Factors associated with ACEQ reporting discrepancy between 6 and 12 months postpartum.

Variable		Unadjusted OR (95% CI)	*p-*value	Adjusted OR^†^ (95% CI)	*p-*value
Baseline ACE					
ACE risk level (ref: Low)					
	Intermediate risk	5.17 (2.90, 9.19)	**<0.001**	**-**	**-**
	High risk	7.33 (3.79,14.18)	**<0.001**	**-**	**-**
Baseline emotional state					
PSS total score		1.02 (0.98, 1.05)	0.335	0.99 (0.96, 1.03)	0.789
PSS level (ref: Low)					
	Moderate – high stress	0.83 (0.53, 1.30)	0.405	0.62 (0.38, 1.03)	0.064
PHQ-9 total score		1.05 (0.99, 1.12)	0.121	0.98 (0.92, 1.05)	0.653
PHQ-9 severity of depressive symptoms (ref: None)					
	Mild – severe depressive symptoms	1.45 (0.88, 2.39)	0.143	0.96 (0.55, 1.67)	0.874

Outcome is ACEQ reporting discrepancy (any change vs. no change between 6 and 12 months). The relationship between baseline ACEQ score and discrepancy was non-linear (significant quadratic term, *p* < 0.001); the continuous linear association is therefore not presented, and baseline ACE is instead characterized using risk categories. ^†^Adjusted models included baseline ACEQ score modeled as a continuous variable with a quadratic term. Sensitivity analyses adjusting for alternative ACEQ specifications are presented in (**Supplementary Table S3**. OR: odds ratio; CI: confidence interval; PSS: Perceived Stress Scale; PHQ-9: Patient Health Questionnaire-9. *p* < 0.05 is shown in bold font.

In bivariate comparisons, mothers with any reporting discrepancy had slightly higher baseline PHQ-9 scores than those with stable reporting (3.0 [1.0, 5.0] vs. 2.0 [0.0, 4.0]; *p* = 0.035), whereas baseline PSS did not differ (12.0 [8.0, 16.0] vs. 11.0 [6.0, 16.0]; *p* = 0.293; **Supplementary Table S2**). Neither PSS nor PHQ-9 was associated with reporting discrepancy in unadjusted or quadratic adjusted models (**Table 4**), and this null association was held under linear adjustment for baseline ACEQ. Under categorical adjustment, however, moderate-to-high stress showed an inverse association with discrepancy (OR: 0.59, 95% CI: 0.35, 0.97; *p* = 0.038; **Supplementary Table S3**). Because baseline ACE is a component of the change outcome, adjusting it induces a statistical artifact rather than revealing the actual effect, and this does not change the conclusion. Thus, while baseline emotional distress was associated with the number of ACEs reported in the mixed-effects models, it was not associated with whether reported ACEQ scores changed between timepoints.

### Factors associated with direction of discrepancy in ACEQ reporting

Among the 150 mothers reporting discrepancy, 78 (52%) reported more ACEs at 12 months (increase), and 72 (48%) reported fewer (decrease). The direction of change varied by baseline ACE risk level (χ^2(4)^ = 61.58; *p* < 0.001; **Figure 2**; **Supplementary Table S4**). Among low-risk mothers (0 ACEs), 78.9% reported stable scores and 21.1% reported increases; decreases were not possible in this group, as scores could not fall below zero (floor effect). Among intermediate-risk mothers (1–3 ACEs), 25.2% showed increases and 32.8% decreases, and among high-risk mothers (4+ ACEs), 28.6% showed increases and 37.7% decreases. Increases occurred at similar rates across risk levels (21 – 29%), whereas the proportion showing decreases rose with baseline ACE risk level (from none among low-risk mothers to 32.8% and 37.7% at intermediate and high risk). In multinomial models (reference = stable), the association between baseline ACE risk level and increases was estimable (**Supplementary Table S5**), whereas the association with decreases could not be estimated, because low-risk mothers could not report a decrease, producing a structural zero in the reference group (floor effect). In an exploratory analysis restricted to mothers with at least one baseline ACE (n = 208), who were able to report a decrease, the association between baseline ACE and direction of change attenuated to non-significance (**Supplementary Table S5**).

**Figure 2:**
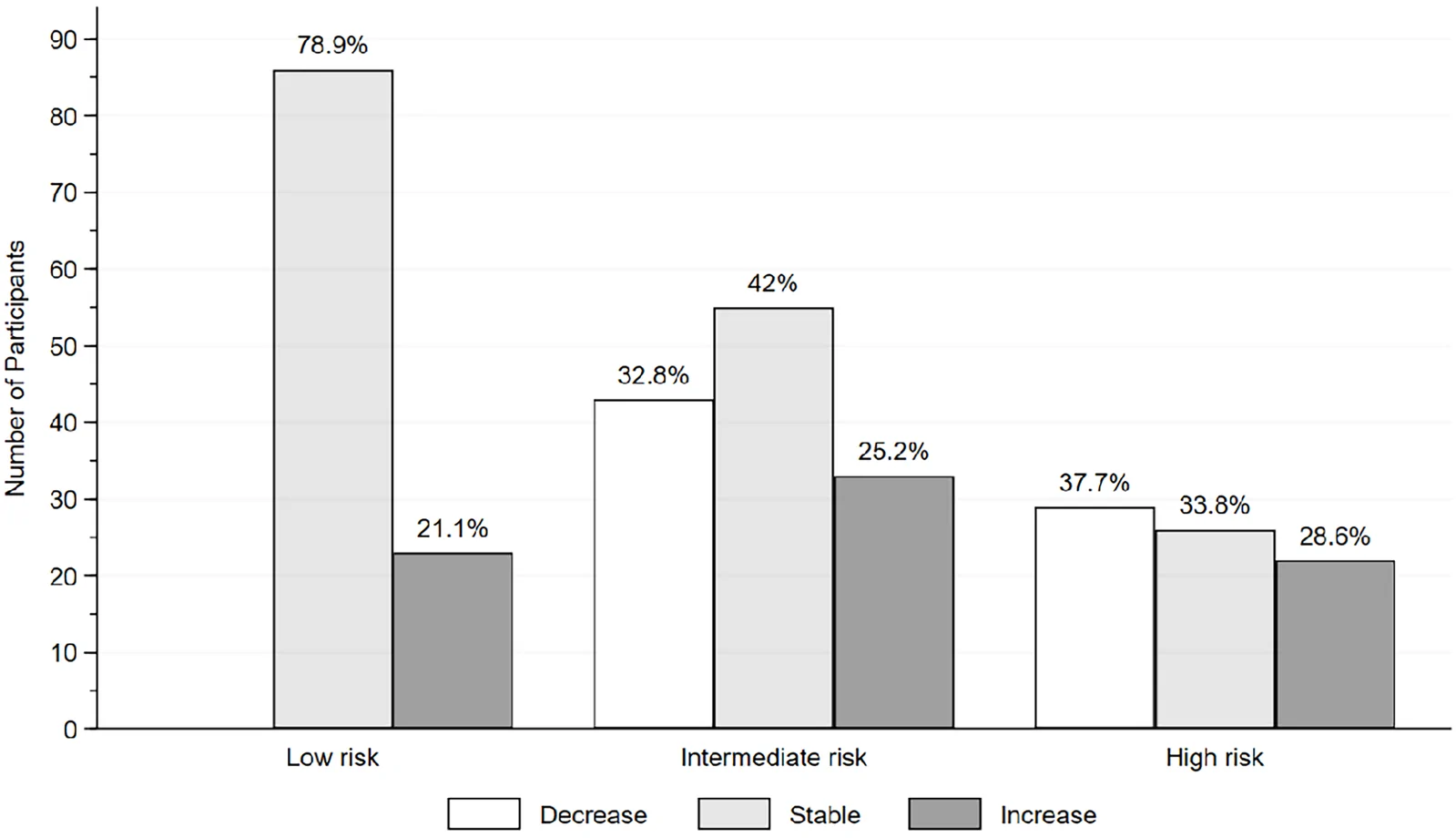
Direction of change in ACEQ reporting between 6 and 12 months, by baseline ACE risk level.

Because the continuous baseline ACEQ score showed a non-linear relationship with direction of change (significant quadratic terms, both *p* < 0.05), it was modeled with a quadratic term in adjusted analyses. Baseline emotional distress was not associated with the direction of change in ACEQ reporting (**Table 5**). In sensitivity analyses adjusting for baseline ACEQ, moderate-to-high stress showed an inverse association with increases (RRR: 0.54, 95% CI: 0.30, 0.97; *p* = 0.041); as in the discrepancy models, this is an artifact of the adjustment; here, regression to the mean. All other distress associations, including every-PHQ-9 estimate, were non-significant (**Table 5**). Model specifications and restricted analysis are presented in **Supplementary Table S5**.

**Table 5:** Factors associated with baseline emotional distress and the direction of change in ACEQ reporting between 6 and 12 months postpartum.

		RRR (95% CI)			
Variable		Increased vs. Stable	*p-*value	Decreased vs. Stable	*p-*value
Unadjusted					
*Baseline emotional state*					
PSS total score		1.00 (0.95, 1.04)	0.825	1.04 (1.00, 1.09)	0.070
PSS level (ref: Low)					
	Moderate – high stress	0.65 (0.37, 1.15)	0.141	1.05 (0.60, 1.83)	0.870
PHQ-9 total score		1.06 (0.98, 1.14)	0.125	1.04 (0.96, 1.12)	0.305
PHQ-9 severity of depressive symptoms (ref: None)		1.55 (0.85, 2.81)	0.150	1.35 (0.73, 2.52)	0.343
	Mild – severe depressive symptoms				
Adjusted for baseline ACEQ^†^					
PSS total score		0.98 (0.94, 1.03)	0.376	1.02 (0.97, 1.07)	0.546
PSS level (ref: Low)					
	Moderate – high stress	0.54 (0.30, 0.97)	**0.041**	0.75 (0.40, 1.39)	0.361
PHQ-9 total score		1.01 (0.94, 1.10)	0.707	0.95 (0.87, 1.03)	0.219
PHQ-9 severity of depressive symptoms (ref: None)					
	Mild – severe depressive symptoms	1.19 (0.63, 2.24)	0.597	0.72 (0.36, 1.46)	0.366

Multinomial logistic regression with stable reporting as the reference outcome. ^†^Adjusted models included baseline ACEQ score modeled as a continuous variable with a quadratic term. The association between baseline ACEQ score and direction of change was non-linear; a categorical ACE risk-level specification could not be estimated for the decrease outcome because low-risk mothers (with no ACEs) could not report a decrease, producing a structural zero in the reference group (floor effect see **Supplementary Table S5**). RRR: relative risk ratio; CI: confidence interval; PSS: Perceived Stress Scale; PHQ-9: Patient Health Questionnaire-9. *p* < 0.05 is shown in bold font.

## Discussion

This study of a moderate-sized cohort of postpartum women provided an opportunity to examine the relative stability of questionnaire-based ELA assessment among caregivers. Analyses revealed that while discrepancies were present among this sample, a majority of participants showed stable scores (within the minimum detectable change threshold) across both timepoints. This is consistent with previous studies, which found that the ACEQ was overall a reliable measure when utilized among broader adult populations (12, 13, 33). Agreement on ACE risk level classification was also substantial (κ = 0.62) supporting the use of categorical risk levels. We found that a change exceeding 3.1 points on the ACEQ (i.e., a change of 4 or more endorsed items in either direction) represented change beyond measurement error, whereas smaller changes fell within the range of measurement variability. This threshold, however, also reflects a limitation: because it corresponds to roughly one-third of the scale range, only larger shifts can be distinguished from measurement error, and only 4.4% of mothers showed change of this magnitude. Because this threshold also approximates the spacing of the ACE risk categories, measurement error alone can reclassify an individual into a different risk category on re-administration. Smaller true changes cannot be reliably detected, an important consideration for any use of the ACEQ to track scores over time. Future studies may consider evaluating characteristics of the few participants who demonstrated change in ACEQ score beyond measurement error, to understand factors associated with real change in reported adversity.

Higher baseline stress and depressive symptoms were associated with higher reported ACEs scores at both timepoints, with the PHQ-9 showing a stronger association than the PSS. Specifically, mothers with mild-to-severe depressive symptoms reported significantly more ACEs than those without symptoms, whereas the difference between moderate-to-high and low stress groups did not reach significance. This is consistent with other studies, such as in Colman et al. (17), which reported that development of mental health conditions, including depression, was associated with an increase in reported ACEs. Because childhood adversity is temporally fixed, this cross-sectional association cannot establish whether adversity contributes to emotional distress or if distress influences recall; clinically, however, distress was not associated with the consistency of reporting: it did not relate to whether scores changed, and its association with reported ACEQ scores did not vary between timepoints (non-significant time × distress interaction). In other words, emotional distress was linked to how many ACEs mothers reported, but not to how consistently they reported them, so it does not undermine the ACEQ’s reliability. Consistent with evidence that ACEs are a risk factor for PPD, ACEQ scores may help to identify post-partum patients who may need additional mental health screening and monitoring.

Baseline emotional state was not significantly associated with reporting discrepancy, despite a modest bivariate association between PHQ-9 and discrepancy status. Discrepancy was instead more common among mothers with higher baseline ACE levels: those at intermediate and high ACE risk had higher odds of any change in reported endorsements rather than low risk mothers. This pattern, however, appears largely structural rather than a substantive effect of adversity on reporting. As the discrepancy outcome is defined relative to the baseline ACEQ score, mothers reporting no baseline adversity are constrained by the floor of the scale and can only remain stable or increase, whereas those with higher baseline scores have greater scope to change in either direction. Notably, 91% of observed discrepancies fell within the measurement-error threshold, indicating that most changes reflected measurement variability rather than true change. Previous studies of emotional state measures have reported mixed findings: Colman et al. (17) reported that individuals with depression were more likely to report ACEQ discrepancies upon a second administration, whereas Frampton et al. (12) found the PHQ-9 was not a significant predictor of ACEQ score change – consistent with our finding that emotional distress was not associated with discrepancy. To the authors’ knowledge, prior ACEQ reliability studies reported overall agreement but did not examine how mother’s baseline score shapes the change seen on re-administration. This study shows that such change is driven largely by the scale floor and regression to the mean, rather than by a true shift in reported adversity.

Given that most mothers reported minimal depressive symptoms, the null association between emotional distress and discrepancy may partly reflect limited power to detect a weak effect; a sample with greater variation in emotional distress would provide a stronger test, though the null regression results argue against a strong effect. Among mothers whose score changed, the direction of change was related to baseline ACEQ score in a manner consistent with regression to the mean: those with higher baseline ACEQ scores more often reported decreases, whereas those at the floor (no baseline adversity) could only increase. Together with the floor effect, this regression-to-the-mean pattern likely accounts for the association between baseline ACE level and both the occurrence and direction of change, rather than an effect of adversity on reporting. In an analysis restricted to mothers with at least one baseline ACE, among whom a decrease was possible, the association between baseline ACEQ and direction of change attenuated to non-significance, indicating that the full-sample pattern was driven largely by the scale’s floor. Consistent with this, the Bland-Altman analysis, which references the mean of the two administrations rather than the baseline ACEQ score and thus avoids this dependence, showed no proportional bias, with agreement unrelated to ACEQ score magnitude.

The current study sample included a broader range of racial/ethnic groups than the original ACEs study, with a majority identifying as Hispanic/Latine. These findings suggest that the ACEQ demonstrates adequate reliability not only among women up to 1 year postpartum but also within a more ethnically and racially diverse sample than the largely White population of European descent in which it was originally established. Future research could examine whether reliability varies across specific racial/ethnic groups.

## Limitations

There are several limitations to the current study. As a self-reported questionnaire, the ACEQ is subject to risk of reporting bias. Given the sensitive nature of the subject matter, participants may underreport or inaccurately report childhood experiences, despite the use of a deidentified format designed to minimize this risk. However, the deidentified format does result in the inability to track item-level responses across timepoints, limiting the ability to assess which specific types of adversity contributed to score changes.

The ACEQ does not capture the severity or chronicity of the adverse childhood experiences, which may impact reporting consistency. Additionally, experiences of emotional distress, specifically depression, prior to parity have been shown to serve as a risk factor for PPD. Therefore, it would be of value to control for maternal history of depression in future studies to determine whether the ACEQ may serve as an additional PPD screening tool among those without a prior history of depression.

Reliability was assessed over a single 6-month interval with two administrations; stability over longer periods or additional timepoints were not evaluated. Both time points were determined by the larger parent study, and future reliability studies should administer assessments prenatally.

Finally, the study population was composed of predominantly middle-to-high income and highly educated mothers, largely due to recruitment through Kaiser Permanente, a main recruitment site for parent study. Due to this limited variation in income and education, we did not examine socioeconomic status as a moderator in the present sample as the limited variation was insufficient power to detect such effects. Future research should examine whether socioeconomic and educational factors moderate ACEQ reliability or the association between ACEs and postpartum outcomes, given that ACE exposure and postpartum depression are known to vary across these contexts.

## Conclusion

 The findings of this study suggest that the deidentified ACEQ is a reliable tool for assessing self-reported ELA among postpartum women during the first year following delivery, with scores remaining consistent across the 6-month interval between administrations. Establishing the ACEQ’s reliability in this population is a necessary step towards future research on the association relationship of maternal ACEs with mental and physical health outcomes during pregnancy and in the postpartum periods. Future research should continue to explore the reliability and utility of the ACEQ during the prenatal and postpartum periods by measuring if the ACEQ can improve postpartum care and strengthen its potential as a standardized perinatal screening tool. If the ACEQ is established as a reliable measure during early postpartum, when PPD onset is most prevalent, the ACEQ may provide critical insight that can help guide maternal clinical care and early interventions for health perturbations linked to ACEs.

## Data Availability

The raw data supporting the conclusions of this article will be made available by the authors, without undue reservation.
