# Supplementary material for: The reliability of the adverse childhood experiences questionnaire for late postpartum use in relation to maternal emotional health
Source: Front Psychiatry. 2026 Oct 19;17:1914194. doi: 10.3389/fpsyt.2026.1914194 (PMC13724549; doi:10.3389/fpsyt.2026.1914194)
Supplement: Supplementary file 1 [file Table1.docx]

| **Table S1**. Pairwise Spearman correlations between baseline maternal emotional distress measures and early life adversity at 6 and 12 months postpartum | | | |
| --- | --- | --- | --- |
|  | ACEs_6 months_ | ACEs_12 months_ | Baseline PHQ-9 |
| Baseline PSS | 0.205^**^ | 0.121^*^ | 0.574^**^ |
| Baseline PHQ-9 | 0.337^**^ | 0.313^**^ | - |
| Spearman correlation coefficients were calculated using all available data (n = 317). ^*^*p* < 0.05, ^**^*p* < 0.001. | | | |
