# Supplementary material for: The reliability of the adverse childhood experiences questionnaire for late postpartum use in relation to maternal emotional health
Source: Front Psychiatry. 2026 Oct 19;17:1914194. doi: 10.3389/fpsyt.2026.1914194 (PMC13724549; doi:10.3389/fpsyt.2026.1914194)
Supplement: Supplementary file 2 [file Table2.docx]

| **Table S2.** Baseline characteristics by ACEQ reporting discrepancy status between 6 and 12 months postpartum | | | | |
| --- | --- | --- | --- | --- |
| Variable | | Any change | No change | *p-*value^‡^ |
|  | | (n = 150) | (n = 167) |  |
| ACEQ total score | | 2.0 [1.0, 4.0] | 0.0 [0.0, 2.0] | **<0.001** |
| ACE risk level | |  |  | **<0.001** |
|  | Low | 23 (15.3%) | 86 (51.5%) |  |
|  | Intermediate | 76 (50.7%) | 55 (32.9%) |  |
|  | High | 51 (34.0%) | 26 (15.6%) |  |
| PSS total score | | 12.0 [8.0, 16.0] | 11.0 [6.0, 16.0] | 0.293 |
| PSS level | |  |  | 0.423 |
|  | Low stress | 94 (62.7%) | 97 (58.1%) |  |
|  | Moderate – high stress | 56 (37.3%) | 70 (41.9%) |  |
| PHQ-9 total score | | 3.0 [1.0, 5.0] | 2.0 [0.0, 4.0] | **0.035** |
| PHQ-9 severity of depressive symptoms | |  |  | 0.163 |
|  | None | 104 (69.3%) | 128 (76.6%) |  |
|  | Mild – severe depressive symptoms | 46 (30.7%) | 39 (23.4%) |  |
| Data are frequency and percentage or median [25^th^, 75^th^ percentiles]. ^‡^Bivariate p-values from chi-square tests (categorical variables) or Wilcoxon rank-sum tests (continuous variables). *p* < 0.05 is shown in bold font. | | | | |
