# Supplementary material for: The reliability of the adverse childhood experiences questionnaire for late postpartum use in relation to maternal emotional health
Source: Front Psychiatry. 2026 Oct 19;17:1914194. doi: 10.3389/fpsyt.2026.1914194 (PMC13724549; doi:10.3389/fpsyt.2026.1914194)
Supplement: Supplementary file 3 [file Table3.docx]

| **Table S3.** Sensitivity analysis: Factors associated with ACEQ reporting discrepancy between 6 and 12 months postpartum | | | |
| --- | --- | --- | --- |
| Variable | | Adjusted OR^†^ (95% CI) | *p-*value |
| **Adjusting for ACEQ Score (linear)** | |  |  |
| PSS total score | | 1.00 (0.96, 1.04) | 0.927 |
| PSS level (ref: Low) | |  |  |
|  | Moderate-high stress | 0.68 (0.42, 1.10) | 0.115 |
| PHQ-9 total score | | 0.99 (0.93, 1.07) | 0.882 |
| PHQ-9 severity of depressive symptoms (ref: None) | |  |  |
|  | Mild – severe depressive symptoms | 1.00 (0.58, 1.73) | 0.992 |
| **Adjusting for ACE Risk Level (categorical)** | |  |  |
| PSS total score | | 0.99 (0.95, 1.03) | 0.639 |
| PSS level (ref: Low) | |  |  |
|  | Moderate – high stress | 0.59 (0.35, 0.97) | **0.038** |
| PHQ-9 total score | | 0.98 (0.92, 1.05) | 0.624 |
| PHQ-9 severity of depressive symptoms (ref: None) | |  |  |
|  | Mild – severe depressive symptoms | 0.99 (0.56, 1.73) | 0.964 |
| Sensitivity analyses adjusted emotional distress associations for categorical ACE risk level or a linear ACEQ term. The relationship between baseline ACEQ score and discrepancy was non-linear (significant quadratic term, p < 0.001). OR: odds ratio; CI: confidence interval; PSS: Perceived Stress Scale; PHQ-9: Patient Health Questionnaire-9. *p* < 0.05 is shown in bold font. | | | |
