# Supplementary material for: The reliability of the adverse childhood experiences questionnaire for late postpartum use in relation to maternal emotional health
Source: Front Psychiatry. 2026 Oct 19;17:1914194. doi: 10.3389/fpsyt.2026.1914194 (PMC13724549; doi:10.3389/fpsyt.2026.1914194)
Supplement: Supplementary file 4 [file Table4.docx]

| **Table S4.** Baseline characteristics by direction of ACEQ reporting change between 6 and 12 months postpartum | | | | | |
| --- | --- | --- | --- | --- | --- |
| Variable | | Decreased | Stable | Increased | *p-*value^‡^ |
|  | | (n = 72) | (n = 167) | (n = 78) |  |
| ACEQ total score | | 3.0 [2.0, 5.0] | 0.0 [0.0, 2.0] | 2.0 [0.0, 4.0] | **<0.001** |
| ACE risk level | |  |  |  | **<0.001** |
|  | Low | 0 (0.0%) | 86 (51.5%) | 23 (29.5%) |  |
|  | Intermediate | 43 (59.7%) | 55 (32.9%) | 33 (42.3%) |  |
|  | High | 29 (40.3%) | 26 (15.6%) | 22 (28.2%) |  |
| PSS total score | | 12.5 [9.0, 17.0] | 11.0 [6.0, 16.0] | 10.0 [7.0, 16.0] | 0.127 |
| PSS level | |  |  |  | 0.266 |
|  | Low stress | 41 (56.9%) | 97 (58.1%) | 53 (67.9%) |  |
|  | Moderate – high stress | 31 (43.1%) | 70 (41.9%) | 25 (32.1%) |  |
| PHQ-9 total score | | 2.5 [1.0, 5.0] | 2.0 [0.0, 4.0] | 3.0 [1.0, 5.0] | 0.111 |
| PHQ-9 severity of depressive symptoms | |  |  |  | 0.310 |
|  | None | 51 (70.8%) | 128 (76.6%) | 53 (67.9%) |  |
|  | Mild – severe depressive symptoms | 21 (29.2%) | 39 (23.4%) | 25 (32.1%) |  |
| Data are frequency and percentage or median [25^th^, 75^th^ percentiles]. ^‡^p-values from chi-square tests (categorical variables) or Kruskal-Wallis tests (continuous variables). *p* < 0.05 is shown in bold font. | | | | | |
