# Supplementary material for: The reliability of the adverse childhood experiences questionnaire for late postpartum use in relation to maternal emotional health
Source: Front Psychiatry. 2026 Oct 19;17:1914194. doi: 10.3389/fpsyt.2026.1914194 (PMC13724549; doi:10.3389/fpsyt.2026.1914194)
Supplement: Supplementary file 5 [file Table5.docx]

| **Table S5.** Associations between baseline ACE and the direction of change in ACEQ reporting: model specifications and restricted (exploratory) analysis | | | | | |
| --- | --- | --- | --- | --- | --- |
|  | | RRR (95% CI) | | | |
|  | | Increased vs. Stable | *p-*value | Decreased vs. Stable | *p-*value |
| Full sample^†^ | |  |  |  |  |
| **Adjusting for ACEQ Score (linear)** | |  |  |  |  |
| PSS total score | | 0.99 (0.94, 1.03) | 0.513 | 1.02 (0.98, 1.07) | 0.336 |
| PSS level (ref: Low) | |  |  |  |  |
|  | Moderate – high stress | 0.58 (0.32, 1.03) | 0.064 | 0.82 (0.45, 1.51) | 0.533 |
| PHQ-9 total score | | 1.02 (0.95, 1.10) | 0.569 | 0.96 (0.88, 1.05) | 0.367 |
| PHQ-9 severity of depressive symptoms (ref: None) | |  |  |  |  |
|  | Mild – severe depressive symptoms | 1.19 (0.63, 2.24) | 0.597 | 0.72 (0.36, 1.46) | 0.366 |
| **Adjusting for ACE Risk Level** | |  |  |  |  |
| PSS total score | | 0.98 (0.94, 1.02) | 0.361 | **-** | **-** |
| PSS level (ref: Low) | |  |  |  |  |
|  | Moderate – high stress | 0.52 (0.29, 0.95) | **0.034** | **-** | **-** |
| PHQ-9 total score | | 1.01 (0.94, 1.10) | 0.734 | **-** | **-** |
| PHQ-9 severity of depressive symptoms (ref: None) | |  |  | **-** | **-** |
|  | Mild – severe depressive symptoms | 1.20 (0.63, 2.27) | 0.579 |  |  |
| Baseline ACE association, restricted to baseline ACE ≥ 1 (n = 208, exploratory) | |  |  |  |  |
| Continuous ACEQ score | | 1.07 (0.92, 1.24) | 0.402 | 1.12 (0.98, 1.29) | 0.100 |
| ACE risk level (high vs. intermediate) | | 1.41 (0.69, 2.88) | 0.345 | 1.43 (0.74, 2.77) | 0.293 |
| Multinomial logistic regression with stable reporting as the reference outcome. In the full sample, the continuous ACEQ score showed a non-linear relationship with direction of change (significant quadratic terms). ^†^The categorical decrease association could not be estimated because low-risk mothers (no ACEs) could not report a decrease, producing perfect separation (floor effect). The exploratory restricted analysis (baseline ACE ≥ 1) included only mothers capable of reporting a decrease; associations attenuated to non-significance, consistent with regression to the mean. RRR: relative risk ratio; CI: confidence interval; PSS: Perceived Stress Scale; PHQ-9: Patient Health Questionnaire-9. *p* < 0.05 is shown in bold font. | | | | | |
